# Age-Related Variations in Body Composition and Metabolic Health: A Cross-Sectional Study in Adults

**DOI:** 10.3390/medicina61111951

**Published:** 2025-10-30

**Authors:** Inga Fomčenko, Inga Bikulčienė, Dovilė Karčiauskaitė, Mykolas Urbonas, Vidmantas Alekna, Virginijus Šapoka

**Affiliations:** 1 Faculty of Medicine, Vilnius University, 01513 Vilnius, Lithuania; 2 Translational Health Research Institute, Faculty of Medicine, Vilnius University, 01513 Vilnius, Lithuania; 3 Antakalnis Outpatient Clinic, 10207 Vilnius, Lithuania

**Keywords:** metabolic health, lipids, fat mass, oxidative stress, sex differences

## Abstract

*Background and Objectives:* Midlife represents a critical window for the emergence of metabolic risk factors. This study aimed to investigate age- and sex-related changes in lipid profiles, body composition, oxidative stress, and fatty acid content. *Materials and Methods:* This cross-sectional study included adults grouped by age: <30, 30–39, and 40–49 years. The assessments covered body composition (fat mass, fat distribution, and lean mass), fasting lipids, inflammation markers measurements, and platelet fatty acids evaluation. *Results:* In total, 169 adults took part in this study (60 men and 109 women), aged 36.30 ± 6.25 years. Fat mass and its regional distribution were higher after age 40, especially in females. In women, fat mass was lower in the thirties and higher again in the forties, while, in men, fat accumulation was progressive. Participants aged 40–49 had a significantly worse metabolic profile than younger individuals. Statistically significant higher total cholesterol, LDL cholesterol, triglycerides, and glucose were shown in the 40–49-years group when compared to younger groups. Malondialdehyde was higher in the 40–49-years vs. 30–39-years group (105.83 vs. 99.72, *p* = 0.034). In women aged 40–49, a more adverse lipid and glycemic profile was observed compared to younger groups. Platelet fatty acids in the 40–49-years group showed higher polyunsaturated fatty acids and ω6 percentages (12.85% vs. 10.14%, *p* = 0.046 and 11.44% vs. 8.79%, *p* = 0.031), including higher linoleic (8.80 ± 5.18 vs. 6.97 ± 5.05, *p* = 0.045), arachidonic (2.64 ± 2.64 vs. 1.82 ± 1.73, *p* = 0.030), and docosahexaenoic (0.61 ± 0.86 vs. 0.31 ± 0.49, *p* = 0.008) acids, when compared to younger groups. Fat mass strongly correlated with insulin resistance, triglycerides, and CRP, and inversely with HDL-C. *Conclusions:* Significant age-related changes in body composition, metabolic biomarkers, and platelet fatty acid profiles occur after the age of 40, with distinct gender-specific patterns. The fifth decade of life is a transitional period characterized by central adiposity, deteriorating metabolic profiles, and altered fatty acid composition, especially in women.

## 1. Introduction

Body composition and metabolic health are key determinants of cardiovascular and metabolic disease risk, which remains a leading cause of morbidity and mortality worldwide [1,2,3,4]. Changes in body fat distribution, particularly increases in central adiposity, have been closely linked to adverse lipid profiles, insulin resistance, and systemic inflammation in adult populations [5]. Age-related increases in fat mass and alterations in lean mass composition contribute significantly to these metabolic disturbances; yet, the timing and nature of these changes remain incompletely characterized, especially in early to mid-adulthood. While numerous longitudinal studies have documented progressive fat accumulation and metabolic dysregulation in older adults [6,7,8,9,10], cross-sectional analyses provide critical image that help clarify age- and sex-specific variations in body composition and metabolism across different stages of adulthood. These data are essential for identifying early patterns that may precede overt metabolic disease. Lipid metabolism, glucose homeostasis, and oxidative stress markers such as malondialdehyde (MDA) have emerged as important metabolic parameters reflecting cardiometabolic risk [11,12,13]. However, the evidence is limited regarding how these metabolic parameters correlate with detailed body composition measures, including the fat mass index (FMI), regional fat depots, and platelet fatty acid composition, in generally healthy young to middle-aged adults. Furthermore, sex differences in these associations and the impact of adiposity categories on metabolic profiles warrant further investigation [14,15]. Therefore, it is relevant to investigate how variations in body composition are associated with key aspects of lipids and glucose metabolism parameters, as well as markers of inflammation and oxidative stress, and the fatty acid composition of platelet membranes.

The aim of this study is to investigate the relationships of age-related variations in body composition and metabolic health in adults.

## 2. Materials and Methods

### 2.1. Study Population

This cross-sectional study was conducted in the outpatient clinic at the National Osteoporosis Center in Vilnius, Lithuania, from May 2023 to June 2024. Inclusion criteria: community-dwelling women and men, age from 18 to 49 years, and voluntary agreement to participate in the study. Exclusion criteria were acute illness and post-surgical follow-ups, chronic cardiometabolic conditions (such as diabetes, hypertension, and heart failure), endocrine disorders (thyroid and adrenal), being in early or well-managed stages, cancer, and using medications affecting body mass or metabolism.

The study was approved by the Vilnius Regional Biomedical Research Ethics Committee (2021/2-1309-787), and everyone was provided with an informed consent form for participation and publication of anonymized results. After the agreement was signed, the medical and family history was evaluated, and clinical examination was performed. Body mass index, total and regional body composition, laboratory analyses, and platelet membrane fatty acid profiles were evaluated for all subjects.

Study participants were divided into three groups according to age: below 30 years, 30–39 years, and 40–49 years.

### 2.2. Body Composition and Anthropometrics

All participants underwent medical history review and clinical examination. After clinical evaluation, weight (kg) was measured using electronic medical scales (Radwag, Radom, Poland), with an accuracy of 50 g. A standard vertical height meter (Harpenden Stadiometer, “Holtain limited”, Crymych, UK) was used to measure height (mm). Body mass index (BMI) was calculated via the following formula: body mass (kg)/height (m^2^). Body composition was assessed using dual-energy X-ray absorptiometry (DXA) (GE Healthcare Lunar iDXA). The results of the body composition measurements (total and regional fat mass and lean mass) were expressed in absolute numbers and percentages (kg, %) [16,17]. Different indicators were calculated: lean mass index (LMI = lean mass (kg)/height (m^2^)), fat mass index (FMI = fat mass (kg)/height (m^2^)), the android-to-gynoid (A/G) ratio (android fat mass (kg) divided by the gynoid fat mass (kg)), and the trunk-to-leg ratio (T/L) (total trunk fat (kg) divided by total leg fat (kg)) [16].

### 2.3. Laboratory Analyses

All blood samples were collected in the early morning (7–9 a.m.) after a 12 h overnight fast. Serum concentrations of total cholesterol, low-density lipoprotein cholesterol (LDL-C), high-density lipoprotein cholesterol (HDL-C), triglycerides (TG), C-reactive protein (CRP), glucose, and insulin were determined using standardized enzymatic and electrochemiluminescence immunoassay methods (Roche Diagnostics, Mannheim, Germany). The homeostatic model assessment for insulin resistance (HOMA-IR) score was calculated via the following formula: glucose × insulin/22.5. Serum malondialdehyde (MDA) concentrations were determined via high-performance liquid chromatography (the commercial kit “Chromsystems Diagnostics by HPLC&LC-MS/MS: Malondialdehyde in Plasma/Serum” (Chromsystems, Gräfelfing, Germany)) according to the manufacturer’s instructions. Determination of human paraoxonase (PON1) concentration in blood serum was performed by enzyme-linked immunosorbent assay (ELISA) using a commercial kit “The Human PON1 ELISA Kit” (Thermo Fisher, Waltham, MA, USA) designed to detect and quantify human PON1. The concentrations of oxidized high-density lipoprotein (OxHDL) and oxidized low-density lipoprotein (OxLDL) in the serum were determined using enzyme-linked immunosorbent assay (ELISA) via the “Human OxHDL ELISA Kit” and “Human OxLDL ELISA Kit” (Abbexa, Cambridge, UK). Lipids were extracted from platelet membranes according to the Folch method [18]. Thin-layer chromatography (TLC; Sil G-25 UV 254) was used to separate the platelet membrane phospholipids [19]. Fatty acid (FA) methyl esters were analyzed via gas chromatography-mass spectrometry (GC-MS) (GCMS-QP2010 Ultra, Shimadzu, Kyoto, Japan). The relative content of each FA was calculated as a percentage of the total FA content (100%) and included saturated fatty acids (SFAs): myristic (C14:0), palmitic (C16:0), and stearic (C18:0) acids; monounsaturated fatty acids (MUFAs): palmitoleic (C16:1ω7), vaccenic (C18:1ω7), oleic (C18:1ω9, OA), and gondoic (C20:1ω9) acids; and polyunsaturated fatty acids (PUFAs). PUFAs were further categorized as omega-3 (ω3) FAs, including α-linolenic (C18:3ω3, ALA), eicosapentaenoic acid (C20:5ω3, EPA), docosapentaenoic (C22:5ω3, DPA), and docosahexaenoic (C22:6ω3, DHA) acids, and omega-6 (ω6) FAs, including linoleic (C18:2ω6, LA) and arachidonic (C20:4 ω6, ARA) acids. The PUFA-to-SFA and omega-3-to-omega-6 (ω3 to ω6) ratios were calculated by dividing the total PUFA content by SFA content and omega-3 by omega-6 FAs, respectively.

### 2.4. Statistical Analysis

Descriptive statistics summarized body composition, laboratory parameters, and thrombocyte-membrane fatty-acid composition. Continuous variables are presented as mean ± standard deviation (SD). Normality was assessed with the Shapiro–Wilk test; because several variables showed variance heterogeneity and group sizes were unequal, Welch’s tests were prespecified for group comparisons. For three or more groups, we used Welch’s ANOVA with Satterthwaite-adjusted degrees of freedom, followed by Games–Howell post hoc comparisons. Associations between fatty acids (FAs) and fat mass index (FMI) were examined using Pearson correlations. Two-sided *p* values < 0.05 were considered statistically significant. Statistical analyses were performed in IBM SPSS Statistics, version 30.0. Figures were created using GraphPad Prism (version 10.0) and Microsoft Excel.

## 3. Results

A total of 182 individuals, aged 18–49 years, who were concerned around their body mass and attended the outpatient clinic National Osteoporosis Center for the evaluation of body composition, were invited to participate in this cross-sectional study. Three subjects did not agree to participate; three were excluded due to acute illness, four persons had hypertension, one was previously diagnosed with diabetes, and two persons reported the use of medications for weight loss. Therefore, 169 subjects were enrolled, 109 women (64.5%) and 60 men (35.5%). The mean age of participants was 36.30 ± 6.25 years, and the youngest and oldest subjects were 20 years and 49 years, respectively. The mean BMI was 27.71 ± 6.30 kg/m^2^. Table 1 presents the body composition, and laboratory parameters, including the thrombocyte fat acid (FA) composition, in all study subjects.

Among the participants, 34.3% had normal weight, 31.4% were overweighted, 30.2% were obese, and 4.1% were underweighted. By FMI, 34.9% had a normal fat amount, 58.6% had excess fat, and 6.5% had a fat deficit. No statistically significant differences in body composition were found between the below 30- and 30–39-years groups. After the age of 40, body mass tended to increase due to the fat mass increase, with statistically significant differences observed between the 30–39- and 40–49-years groups (78.33 kg vs. 87.6 kg, *p* = 0.002). The FMI, total fat mass, trunk fat mass, android, gynoid, leg, and arm fat mass were all significantly higher in the 40–49-years group than in the 30–39-years group. Total, trunk, and android fat percentages were statistically significantly higher in the 40–49-years group compared to the 30–39-years group. The A/G ratio and trunk-to-leg fat ratio were higher in the 40–49-years group compared to both the below-30-years group and the 30–39-years group. The LMI and trunk lean mass were statistically significantly higher in the 40–49-years group compared to the 30–39-years group.

**Figure 1:**
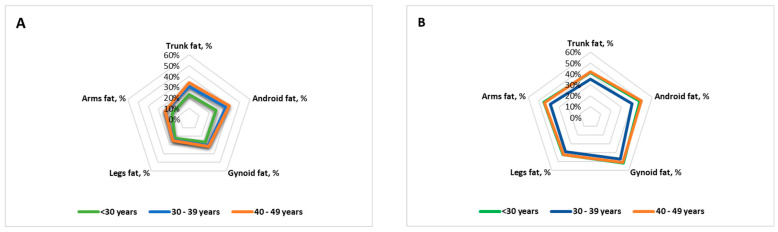
Distribution of regional body fat of male (**A**) and female (**B**) participants by age groups.

The distribution of the regional body fat of male and female participants by age groups is shown in Figure 1.

The body composition characteristics did not differ significantly between males in the 30–39- and 40–49-years groups, and in females in the <30-years- and 40–49-years groups.

When analyzing body composition changes over the years separately for male and female participants, different patterns emerged, as shown in Table 2.

As shown, in males, total body mass and fat mass increase steadily with age. The FMI, total fat mass, trunk fat mass, and android fat mass were significantly higher in the 40–49-years group than in the <30-years group compared to the <30-years group, and the 30–39-years group subjects had statistically significantly higher trunk fat percentage (+7.48%), android fat percentage (+9.26%), and arm fat percentage (+6.60%). In the 40–49-years group, the trunk fat percentage was higher by +11.03%, the android fat percentage by +13.06%, and the arm fat percentage by +6.99%. The A/G ratio and trunk-to-leg fat ratio were higher in the 30–39-years group and in the 40–49-years group compared to the <30-years group.

In females, body composition changes were more variable. A statistically significant lower total body mass, total lean mass, total fat mass, BMI, FMI, trunk lean mass, trunk fat mass, android fat mass, gynoid fat mass, and leg fat mass were observed in the 30–39-years group compared to both the <30-years and 40–49-years groups. Comparing the 30–39-years group with the <30-years group, statistically significant decreases were observed in body fat percentage (−4.94%), trunk fat percentage (−6.39%), android fat percentage (−8.07%), gynoid fat percentage (−4.56%), leg fat percentage (−3.6%), and arm fat percentage (−6.05%). With advancing age, fat mass was regained: compared to the 30–39-years group, the 40–49-years group showed statistically significant increases in body fat percentage (5.09%), trunk fat percentage (6.98%), android fat percentage (8.75%), gynoid fat percentage (3.75%), leg fat percentage (3.29%), and arm fat percentage (5.27%). The A/G ratio and trunk-to-leg fat ratio were higher in in the 40–49 years-group compared to the 30–39-years group.

Table 3 shows the characteristics of metabolic biomarkers and thrombocyte-membrane fatty acids in different age groups.

The laboratory test results showed a statistically significant increase in total cholesterol, LDL cholesterol, triglycerides, and glucose in the 40–49-years group compared to both the 30–39-years and below-30-years groups. MDA was also higher in the 40–49-years group vs. the 30–39-years group (105.83 vs. 99.72, *p* = 0.034). In thrombocyte-membrane fatty acids, the 40–49-years group showed higher PUFAs and ω6 percentages (12.85% vs. 10.14%, *p* = 0.046 and 11.44% vs. 8.79%, *p* = 0.031, respectively), including an increase in C18:2ω6 LA (8.80 ± 5.18 vs. 6.97 ± 5.05, *p* = 0.045), C20:4ω6 ARA (2.64 ± 2.64 vs. 1.82 ± 1.73, *p* = 0.030), and C22:6ω3 DHA (0.61 ± 0.86 vs. 0.31 ± 0.49, *p* = 0.008), with no significant differences in ω3, MUFA, or SFA compared to the 30–39-years group (Table 3). C20:5ω3 EPA was higher in the 40–49-years group compared to the <30-years group (0.52 ± 0.47 vs. 0.33 ± 0.25, *p* = 0.040, respectively).

When analyzing body laboratory biomarkers and thrombocyte-membrane FA composition changes over the years separately for male and female participants, different patterns emerged. In males, no statistically significant differences were found in the lipid profile, glycemic indices, inflammatory/oxidative markers, or thrombocyte FA composition (SFAs, MUFAs, and PUFAs; and ω-3/ω-6 and their ratios) across the <30-years, 30–39-years, and 40–49-years group. The only exception was C22:6ω3 DHA, which was higher in the 40–49-years group compared to the 30–39-years group (0.76 ± 1.14% vs. 0.28 ± 0.32%, *p* = 0.035). Among females, aged 40–49 years, the more adverse lipid profile was found: LDL-C was higher compared to both the <30-years and 30–39-years groups, triglycerides were higher, and HDL-C was lower compared to the 30–39-years group. Glucose was higher compared to both younger groups. In thrombocyte-membrane FA, the 40–49-years group had higher PUFAs, and higher ω-6, with lower SFAs compared to 30–39-years group. Notably, C18:1ω9 OA (8.35 ± 3.04 vs. 6.40 ± 3.59, *p* = 0.020), and C18:2ω6 LA (8.87 ± 3.97 vs. 6.70 ± 4.37, *p* = 0.025) were higher in the 40–49-years group compared to the 30–39-years group.

**Figure 2:**
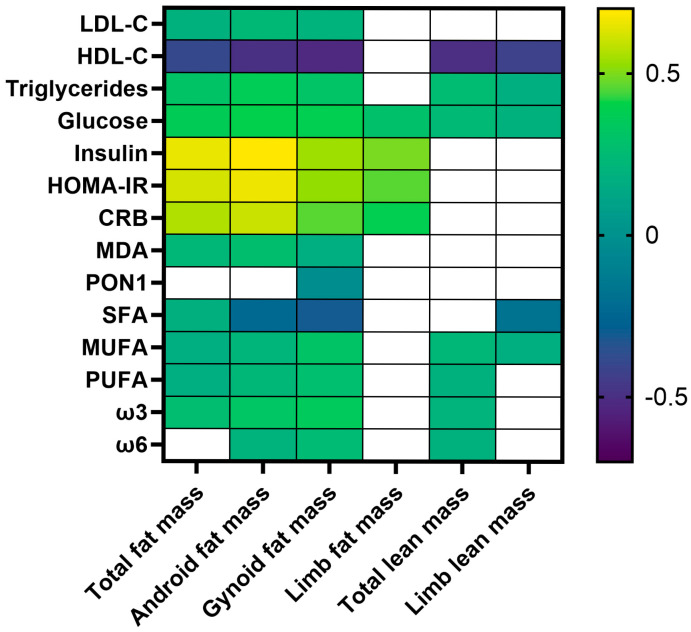
Correlations between body composition and laboratory markers and thrombocyte fatty acids. Pearson correlation coefficients: white cells denote non-significant correlations.

We examined the associations between the total and regional fat and lean mass and metabolic markers and thrombocyte FA using Pearson correlations. Android and gynoid fat mass showed strong inverse correlations with HDL-C (down to r = −0.53), while total fat mass had a moderate inverse correlation with HDL-C (r = −0.39). LDL-C showed weak positive correlations with total, android, and gynoid fat (r = 0.19–0.23). Triglycerides correlated moderately positively with total and regional fat mass (r = 0.30–0.37). Total, android, and gynoid fat mass correlated strongly with insulin (r = 0.54–0.69) and HOMA-IR (r = 0.52–0.66), and moderately with glucose (up to r = 0.39) (Figure 2).

CRB correlated strongly with total and android fat (r = 0.56–0.60) and moderately with gynoid and limb fat (r = 0.39–0.46). MDA showed weak but significant correlations with fat mass. In the thrombocyte FA composition, MUFAs, PUFAs, and ω-3 were positively related to total and regional fat. Total lean mass correlated strongly inversely with HDL-C (r = −0.50); the correlations with triglycerides, glucose, MUFAs, PUFAs, ω-3, and ω-6 were positive but weak.

## 4. Discussion

This study found that body composition and metabolic changes in adults aged 18–49 are most pronounced after age 40, especially in women, with earlier decades showing little difference between age groups. This study fills a critical gap by identifying early, sex-specific metabolic shifts in individuals in their 40s. Its integrated analysis of body composition, metabolic biomarkers, oxidative stress, and platelet fatty acids in a cross-sectional design across younger age groups sets it apart from prior research focused predominantly on older or high-risk populations. The large-scale cross-sectional study by Mohamadzadeh et al. reported that fat mass (FM), body fat percentage, and waist circumference increased significantly from ages 20–29 to 30–39 in both sexes, but the most marked changes —especially declines in lean mass (LM) and increases in FM—became evident after age 40 [20]. This may suggest that the fourth decade represents a metabolic transition period where physiological changes accelerate [21]. A recent Briand et al. cross-sectional analysis using segmented regression identified “break points” in body composition by age. For females, the LM decline could begin earlier (from 31 years), but more substantial alterations, including FM increases and further LM loss, typically occur around 40 years. In males, robust declines in LM generally start after 55, but FM shows a trend towards an increase with age [22]. The supporting epidemiological research has shown that metabolic rate, basal energy expenditure, and fat-free mass are largely stable from ages 20 to 60, with an absence of significant shifts in adulthood except after the fourth decade of life [23]. In our study, the 40–49-years group, compared to the 30–39-years group, showed significantly higher total body mass primarily due to an increased FM rather than LM. Similar results were shown in the cross-sectional study by Barbao et al. which indicated that FM and body fat percent increased with age in both sexes. Ponti et al. also reported that aging is characterized by significant changes in body composition, including an increase in FM, particularly visceral fat, and a decrease in LM [24]. The preferential accumulation of central adiposity, evidenced by increased android FM, trunk FM, and elevated android-to-gynoid (A/G) and trunk-to-leg (T/L) fat ratios in the 40–49-years group, compared to younger groups, aligns with the established literature on age-related changes in fat distribution [25,26]. This pattern is particularly concerning given the well-documented association between central adiposity and metabolic dysfunction, cardiovascular disease risk, and insulin resistance [27,28].

In our study, men showed a steady increase in total body mass and FM with age, and a tendency to store fat in the abdominal region, which may be attributed to hormonal influences, particularly declining testosterone levels with age [29,30]. The progressive increase in A/G and T/L fat ratios with advancing age in men supports multiple previous cross-sectional studies indicating that aging is associated with a shift toward more metabolically harmful fat distribution patterns [25,31,32,33]. Women showed a more complex, U-shaped pattern of body composition changes: body mass and FM were lowest in the 30–39-years group compared to younger and older groups. This nadir could reflect lifestyle factors such as increased physical activity, career-related stress, or dietary changes during the peak reproductive years [14,34]. Several studies using age-period-cohort modelling demonstrate that, while body fat and BMI usually increase steadily through adulthood in longitudinal data, cross-sectional immersion in certain age brackets may reflect that a specific cohort has, on average, different dietary practices, physical activity levels, or socioeconomic backgrounds compared to older or younger cohorts. Educational attainment, public health messaging, or shifting societal norms could produce such cohort-specific trends [35,36]. The subsequent increase in FM in the 40–49-years group, compared to younger age groups, likely corresponds to the onset of perimenopause and declining estrogen levels, which are known to promote central fat accumulation and reduce the metabolic rate [37,38].

Our study results demonstrated that individuals aged 40–49 had a significantly worse lipid profile—higher total cholesterol, LDL-C, and TG—compared to younger groups. This metabolic decline was more pronounced in women: while men showed minimal lipid changes across age groups, women aged 40–49 had significantly higher LDL-C, TG, and glucose levels, and lower HDL-C than younger women. Prior research aligns with these findings: Wenner et al. reported that the lipid profile worsening begins in women in their forties [39]; the ERMA study also showed that menopausal status (which broadly overlaps with the forty-to-fifty transition) independently predicts increases in total cholesterol, LDL-C, and TG above what age alone predicts [40]. Few cross-sectional studies focused on perimenopausal status (late forties) indicated that the already-in-perimenopause lipid profiles are worse than in younger age groups [41,42]. The massive cross-sectional dataset (over a million patients] by Swiger et al. found that, before midlife (twenties through fifties), women have lower LDL-C than men, but, after 50 years, women gradually surpass men’s LDL-C levels [43]. Although not all studies have isolated the 40–49-years bracket, the evidence consistently supports age-related lipid worsening in women, emerging around the perimenopausal years.

In this study, we demonstrated the significant increase in malondialdehyde (MDA) levels in the 40–49-years group compared to the 30–39-years group, which could indicate increased oxidative stress with advancing age. These observations are in line with the free radical theory of aging [44] and prior evidence that oxidative biomarkers, such as MDAs, increase with age, with sharper changes around menopause [15,45].

Our study results indicated the age-related remodelling of platelet-membrane fatty acids, characterized by higher total PUFAs and ω-6 PUFAs in the 40–49-years group, compared to younger groups. Specifically, increases in linoleic acid (C18:2ω6 LA), arachidonic acid (C20:4ω6 ARA), and docosahexaenoic acid (C22:6ω3 DHA) were observed. Moreover, women showed more pronounced changes in FA composition with age in different groups compared to men. The prior scientific literature data are heterogeneous. Otsuka et al. reported age-related increases in serum EPA/DHA with an overall decline in ARA; however, among women in their forties, serum ARA correlated positively with dietary ARA intake [46]. Another study found that plasma DHA increased with age, whereas LA decreased [47]. Aiello et al. delineated complex blood ω-3, ω-6 and oxidative stress/inflammation relationships across the lifespan: a higher ω-3 status was associated with lower oxidative stress in adult women, whereas total ω-3 and ω-6 were positively related to MDA in older strata [48]. Platelet FA composition studies tended to report decreases in LA and sometimes ARA with age [49,50]. The observed rise in platelet-membrane ω-6 PUFAs with age can be considered as a potential pro-inflammatory shift. Mechanistically, ω-6 PUFAs (especially ARA) serve as precursors to pro-inflammatory lipid mediators (eicosanoids) including prostaglandins, thromboxanes, and leukotrienes. These mediators are integral in driving low-grade chronic inflammation and have been implicated in the increase in systemic inflammation seen in older adults. Dietary patterns substantially modulate the membrane composition of fatty acids: the excessive intake of ω-6-rich oils and foods results in the accumulation of LA and ARA within cell and platelet membranes [51,52]. Thus, our findings may, in part, reflect dietary habits common in midlife.

Analyzing body composition and metabolic associations, we found strong positive correlations between FM (particularly android and gynoid fat) and markers of insulin resistance. Chen et al. also found that T/L and A/G fat ratios correlate positively with HOMA IR [53]; and Pinnick et al. [54] reported positive correlations between android FM and insulin, HOMA IR, and TG. Women with android obesity had significantly higher fasting insulin and HOMA-IR compared to gynoid obesity and lean controls, as demonstrated in the study by M. Orbetzova et al. [55]. In normal-weight men with insulin resistance, android fat and total fat were significantly higher compared to insulin-sensitive normal-weight men. Gynoid fat was also higher but the android/gynoid ratio was elevated [56]. The inverse correlations between regional fat mass and HDL-C cholesterol found in this study highlighted the detrimental effects of excess adiposity on cardioprotective lipid profiles. Interestingly, total lean mass also showed an inverse correlation with HDL-C, which may reflect the complex interplay between body composition components, age, and metabolic health [57,58,59]. Our results show the strong positive correlations between FM and CRB, which reinforces the role of adipose tissue as an active endocrine organ that promotes systemic inflammation [60]. Multiple cross-sectional studies likewise report strong positive associations between total and regional fat and CRP, supporting that greater, particularly central, adiposity is accompanied by elevated CRP [61,62,63].

Our study has several strengths. It provides a comprehensive assessment of body composition using DXA, which is considered the gold standard for body composition analysis. The inclusion of thrombocyte-membrane FA analysis adds a novel dimension to understanding age-related metabolic changes. The sex-stratified analyses provide valuable insights into the differential effects of aging on men and women.

Several limitations should be acknowledged. The relatively small sample sizes in age subgroups, particularly for sex-stratified analyses, may limit the generalizability of findings. Additionally, the age range was limited to 18–49 years, precluding the assessment of changes in older adults where more dramatic alterations might be expected.

To conclude, our findings identify the 40-to-49-years period as a critical period for metabolic health deterioration, compared to a younger age, particularly in women. This has important implications for preventive healthcare strategies, suggesting that interventions targeting body composition and metabolic health should start to be intensified during this period. The distinct sex-specific patterns observed in this study emphasize the need for tailored approaches to health assessment and intervention. Women may benefit from closer monitoring during the perimenopausal transition, while men may require earlier intervention for central adiposity prevention. The strong correlations between regional fat distribution and metabolic markers support the use of body composition assessment as a valuable tool for cardiovascular and metabolic risk stratification.

## 5. Conclusions

According to our data, significant age-related changes in body composition, metabolic biomarkers, and platelet fatty acid profiles occur after the age of 40, with distinct gender-specific patterns. The fifth decade of life is a transitional period characterized by central adiposity, deteriorating metabolic profiles, and altered fatty acid composition, especially in women. The strong correlations between regional fat distribution and metabolic markers emphasize the importance of body composition assessment in clinical practice.

## Figures and Tables

**Table 1:** Descriptive characteristics of the body composition, metabolic biomarkers, and thrombocyte-membrane fatty-acids composition of the participants.

Body Composition	Mean ± SD	Biomarkers	Mean ± SD
Total body mass, kg	82.29 ± 17.17	Total cholesterol, mmol/L	4.93 ± 0.87
Total lean mass, kg	51.33 ± 11.51	LDL-cholesterol, mmol/L	3.12 ± 0.83
Total fat mass, kg	28.13 ± 12.27	HDL-cholesterol, mmol/L	1.55 ± 0.41
Trunk lean mass, kg	22.32 ± 4.87	Triglycerides, mmol/L	1.13 ± 0.70
Trunk fat mass, kg	13.61 ± 7.84	Glucose, mmol/L	5.36 ± 0.50
Android fat mass, kg	2.50 ± 1.49	Insulin, mU/L	10.52 ± 6.99
Gynoid fat mass, kg	5.39 ± 2.26	HOMA-IR	2.58 ± 1.94
Legs lean mass, kg	19.17 ± 4.53	CRB, mg/L	1.90 ± 2.66
Legs fat mass, kg	10.32 ± 3.97	Oxidized LDL, ng/mL	294.55 ± 148.61
Arms lean mass, kg	6.12 ± 2.38	Oxidized HDL, ng/mL	35.68 ± 52.82
Arms fat mass, kg	3.14 ± 1.33	MDA, ng/L	101.01± 19.02
Total fat, %	34.58 ± 10.31	PON1, mcg/L	10.75 ± 6.66
Trunk fat, %	35.53 ± 12.19	SFA, %	77.29 ± 12.93
Android fat, %	41.69 ± 14.19	MUFA, %	11.46 ± 6.20
Gynoid fat, %	42.55 ± 11.70	PUFA, %	11.12 ± 7.42
Legs fat, %	34.77 ± 10.25	PUFA to SFA ratio	0.17 ± 0.15
Arms fat, %	34.76 ± 12.85	ω3 fatty acids, %	1.46 ± 1.14
Fat mass index, kg/m^2^	9.44 ± 4.41	ω6 fatty acids, %	9.76 ± 6.77
Lean mass index, kg/m^2^	16.81 ± 2.68	ω3-to-ω6 ratio	0.22 ± 0.24
Android-to-gynoid ratio	0.45 ± 0.17		
Trunk-to-leg ratio	1.31 ± 0.58		

LDL—low-density lipoprotein; HDL—high-density lipoprotein; HOMA-IR—homeostatic model assessment for insulin resistance; CRB—C-reactive protein; MDA—malondialdehyde; PON1—human paraoxonase; SFAs—saturated fatty acids; MUFAs—monounsaturated fatty acids; PUFAs—polyunsaturated fatty acids; ω3—omega-3 fatty acids; ω6—omega-6 fatty acids; SD—standard deviation.

**Table 2:** Anthropometrics and body composition characteristics of the participants by age groups.

Characteristic	<30 Years	30–39 Years	40–49 Years	p1	p2	p3
**Males**						
Total body mass, kg	88.01 ± 12.75	89.88 ± 14.59	92.92 ± 11.39	0.703	0.346	0.432
Total lean mass, kg	66.24 ± 11.31	62.75 ± 9.53	63.21 ± 7.43	0.303	0.399	0.861
Total fat mass, kg	18.30 ± 7.49	23.82 ± 10.47	26.36 ± 8.49	0.114	**0.031**	0.354
Trunk lean mass, kg	27.70 ± 4.44	26.78 ± 4.01	27.17 ± 3.26	0.516	0.721	0.731
Trunk fat mass, kg	8.34 ± 4.01	12.82 ± 7.76	14.81 ± 6.39	0.079	**0.018**	0.316
Android fat mass, kg	1.49 ± 0.92	2.35 ± 1.41	2.72 ± 1.22	0.071	**0.017**	0.331
Gynoid fat mass, kg	3.45 ± 1.82	4.04 ± 1.68	4.17 ± 1.19	0.301	0.240	0.787
Legs lean mass, kg	24.67 ± 5.32	23.31 ± 4.62	23.17 ± 3.24	0.395	0.378	0.914
Legs fat mass, kg	6.98 ± 2.99	7.41 ± 2.16	7.70 ± 1.73	0.592	0.397	0.644
Arms lean mass, kg	9.66 ± 1.93	8.46 ± 2.33	8.59 ± 1.40	0.107	0.174	0.824
Arms fat mass, kg	1.91 ± 0.81	2.46 ± 0.95	2.64 ± 0.75	0.091	0.034	0.459
Total fat, %	21.53 ± 7.70	26.86 ± 8.24	29.02 ± 7.29	0.068	**0.017**	0.346
Trunk fat, %	22.93 ± 9.43	30.41 ± 10.13	33.97 ± 10.12	**0.046**	**0.006**	0.224
Android fat, %	27.00 ± 13.06	36.26 ± 11.54	40.06 ± 12.38	**0.040**	**0.007**	0.279
Gynoid fat, %	26.43 ± 10.29	30.72 ± 7.53	31.98 ± 6.46	0.133	0.068	0.573
Legs fat, %	21.88 ± 7.85	24.36 ± 7.00	25.01 ± 5.01	0.305	0.222	0.732
Arms fat, %	16.65 ± 6.99	23.25 ± 9.90	23.64 ± 6.59	**0.038**	**0.038**	0.872
Body mass index, kg/m^2^	25.18 ± 4.18	27.49 ± 6.20	28.50 ± 3.75	0.229	0.104	0.502
Fat mass index, kg/m^2^	5.28 ± 2.35	7.10 ± 3.03	8.03 ± 2.61	0.080	**0.014**	0.257
Lean mass index	18.90 ± 3.33	18.76 ± 2.54	19.19 ± 2.17	0.880	0.775	0.566
Android-to-gynoid ratio	0.42 ± 0.11	0.56 ± 0.16	0.63 ± 0.18	**0.028**	**0.002**	0.120
Trunk-to-leg ratio	1.22 ± 0.30	1.65 ± 0.59	1.88 ± 0.66	**0.047**	**0.005**	0.178
**Females**						
Total body mass, kg	82.64 ± 18.70	71.91 ± 17.04	84.56 ± 14.20	**0.016**	0.683	**0.001**
Total lean mass, kg	45.70 ± 6.19	42.82 ± 5.62	47.12 ± 4.69	**0.050**	0.363	**<0.001**
Total fat mass, kg	34.34 ± 13.87	26.62 ± 12.76	34.81 ± 10.97	**0.022**	0.893	**0.003**
Trunk lean mass, kg	20.21 ± 2.88	18.51 ± 2.76	21.39 ± 2.95	**0.027**	0.146	**<0.001**
Trunk fat mass, kg	16.24 ± 9.02	11.62 ± 7.62	16.92 ± 7.72	**0.031**	0.763	**0.003**
Android fat mass, kg	2.97 ± 1.72	2.10 ± 1.45	3.19 ± 1.48	**0.032**	0.617	**0.001**
Gynoid fat mass, kg	7.07 ± 2.23	5.38 ± 2.16	6.93 ± 1.89	**0.003**	0.811	**0.001**
Legs lean mass, kg	17.36 ± 2.80	16.28 ± 2.21	17.38 ± 2.20	0.085	0.970	**0.031**
Legs fat mass, kg	13.17 ± 3.82	10.83 ± 3.89	12.96 ± 3.37	**0.020**	0.845	**0.009**
Arms lean mass, kg	4.61 ± 1.02	4.67 ± 0.90	4.81 ± 0.86	0.813	0.457	0.492
Arms fat mass, kg	3.87 ± 1.27	3.20 ± 1.52	3.88 ± 1.10	0.067	0.989	**0.024**
Total fat, %	41.38 ± 8.19	36.43 ± 9.50	41.52 ± 6.62	**0.030**	0.951	**0.006**
Trunk fat, %	41.46 ± 11.30	35.07 ± 13.15	42.04 ± 9.66	**0.045**	0.863	**0.008**
Android fat, %	48.70 ± 12.08	40.63 ± 15.66	49.38 ± 10.43	**0.028**	0.861	**0.004**
Gynoid fat, %	51.76 ± 6.11	47.21 ± 8.30	50.96 ± 5.31	**0.018**	0.692	**0.016**
Legs fat, %	42.50 ± 5.90	38.90 ± 7.25	42.19 ± 5.23	**0.038**	0.863	**0.020**
Arms fat, %	44.93 ± 9.15	38.88 ± 9.97	44.15 ± 5.80	**0.010**	0.753	**0.006**
Body mass index, kg/m^2^	29.53 ± 7.50	25.76 ± 6.58	30.36 ± 5.94	**0.033**	0.658	**0.002**
Fat mass index, kg/m^2^	11.90 ± 4.87	9.46 ± 4.65	12.13 ± 3.84	**0.042**	0.852	**0.007**
Lean mass index	15.84 ± 2.29	15.14 ± 1.90	16.41 ± 1.61	0.167	0.292	**0.002**
Android-to-gynoid ratio	0.39 ± 0.12	0.35 ± 0.13	0.44 ± 0.14	0.244	0.167	**0.001**
Trunk-to-leg ratio	1.16 ± 0.40	0.99 ± 0.39	1.31 ± 0.55	0.144	0.273	**0.002**

Welch’s ANOVA, followed by Games–Howell post hoc comparisons, was used for calculation; p1—comparing <30-years and 30–39-years age groups; p2—comparing <30-years and 40–49-years age groups; p3—comparing 30–39-years and 40–49-years age groups. Bold text—*p* < 0.05.

**Table 3:** Laboratory characteristics of the participants stratified by age groups.

Characteristic	<30 Years, N = 28	30–39 Years, N = 82	40–49 Years, N = 53	p1	p2	p3
Total cholesterol, mmol/L	4.68 ± 1.07	4.85 ± 0.88	5.18 ± 0.68	0.355	**0.014**	**0.033**
LDL cholesterol, mmol/L	2.88 ± 0.94	3.05 ± 0.85	3.36 ± 0.66	0.346	**0.013**	**0.031**
HDL cholesterol, mmol/L	1.63 ± 0.49	1.59 ± 0.43	1.45 ± 0.31	0.645	0.063	0.058
Triglycerides, mmol/L	0.99 ± 0.43	1.04 ± 0.59	1.34 ± 0.91	0.711	**0.029**	**0.015**
Glucose, mmol/L	5.25 ± 0.44	5.30 ± 0.47	5.53 ± 0.53	0.620	**0.013**	**0.007**
Insulin, mU/L	10.40 ± 7.12	10.19 ± 6.29	11.09 ± 7.98	0.891	0.676	0.469
HOMA-IR	2.45 ± 1.71	2.46 ± 1.65	2.84 ± 2.42	0.964	0.388	0.276
CRB, mg/L	2.07 ± 2.77	1.82 ± 2.90	1.92 ± 2.23	0.680	0.816	0.840
Oxidized LDL, ng/mL	272.37 ± 146.85	289.31 ± 152.85	314.97 ± 143.16	0.617	0.242	0.350
Oxidized HDL, ng/mL	63.84 ± 121.71	28.71 ± 14.54	31.60 ± 12.13	**0.003**	**0.011**	0.761
MDA, ng/L	95.76 ± 21.55	99.72 ± 15.74	105.83 ± 21.38	0.882	0.085	**0.034**
PON1, mcg/L	12.02 ± 13.02	10.69 ± 4.69	10.52 ± 3.63	0.375	0.244	0.659
SFA, %	77.05 ± 13.21	79.13 ± 12.15	74.60 ± 13.71	0.470	0.427	0.054
MUFA, %	12.17 ± 7.31	10.73 ± 6.06	12.19 ± 5.75	0.302	0.985	0.196
PUFA, %	10.78 ± 6.39	10.14 ± 7.03	12.85 ± 8.33	0.699	0.243	**0.046**
PUFA-to-SFA ratio	0.16 ± 011	0.15 ± 0.15	0.20 ± 0.17	0.795	0.248	0.068
ω3 fatty acids, %	1.37 ± 0.78	1.35 ± 1.03	1.68 ± 1.41	0.924	0.248	0.104
ω6 fatty acids, %	9.41 ± 5.86	8.79 ± 6.49	11.44 ± 7.42	0.683	0.206	**0.031**
ω3-to-ω6 ratio	0.26 ± 0.33	0.23 ± 0.26	0.18 ± 0.13	0.665	0.187	0.229

Welch’s ANOVA, followed by Games–Howell post hoc comparisons, was used for calculation; p1—comparing <30-years and 30–39-years age groups; p2—comparing <30-years and 40–49-years age groups; p3—comparing 30–39-years and 40–49-years age groups. Bold text—*p* < 0.05. LDL—low-density lipoprotein; HDL—high-density lipoprotein; HOMA-IR—homeostatic model assessment for insulin resistance; CRB—C-reactive protein; MDA—malondialdehyde; PON1—human paraoxonase; SFAs—saturated fatty acids; MUFAs—monounsaturated fatty acids; PUFAs—polyunsaturated fatty acids; ω3—omega-3 fatty acids; ω6—omega-6 fatty acids.

## Data Availability

The dataset generated and/or analyzed during the current study is available from the corresponding author upon reasonable request.

## Abbreviations

The following abbreviations are used in this manuscript:

FA	fatty acid
CRP	C-reactive protein
HDL-C	high-density lipoprotein cholesterol
LDL-C	low-density lipoprotein cholesterol
TG	triglycerides
OxHDL	oxidized HDL
OxLDL	oxidized LDL
MDA	malondialdehyde
PON1	human paraoxonase
SFA	Saturated fatty acid
MUFA	monounsaturated fatty acid
PUFA	polyunsaturated fatty acids
BMI	body mass index
DXA	dual-energy X-ray absorptiometry
FM	fat mass
FMI	fat mass index
LM	lean mass
LMI	lean mass index
A/G	android to gynoid ratio
T/L	trunk-to-leg ratio
HOMA-IR	Homeostatic Model Assessment for Insulin Resistance
ELISA	enzyme-linked immunosorbent assay
ALA	α-linolenic acid
EPA	eicosapentaenoic acid
DPA	docosapentaenoic acid
DHA	docosahexaenoic acid
OA	oleic acid
LA	linoleic acid
ARA	arachidonic acid
SD	standard deviation
GC-MS	gas chromatography-mass spectrometry
ANOVA	Analysis of variance
